# Hospital Needs and Hospital Appeals

**Published:** 1924-12

**Authors:** 


### HOSPITAL NEEDS AND HOSPITAL APPEALS.

### HOSPITALS WITH 150 BEDS AND UPWARDS.

**BROMPTON HOSPITAL FOR CONSUMPTION AND DISEASES OF THE CHEST.**—With its sanatoria and convalescent home at Chobham Ridges, this hospital has nearly 500 beds, all of which are constantly occupied. During 1923, 3,018 in-patients and 33,619 out-patients received treatment at this institution. The annual expenditure is over £75,000, of which only £5,000 is assured. Donors of £52 10s. and annual subscribers of £5 5s. become Governors, and may recommend one in-patient and eight out-patients every year. The Secretary, Mr. Frederick Wood, is making a special appeal for new annual subscribers, and sincerely hopes that the public will respond generously. Contributions may be sent to him at the Hospital for Consumption, Fulham Road, S.W. 3.

**CHARING CROSS HOSPITAL.**—This Hospital is situated right in the heart of the great Metropolis, and every year treats over 100,000 casualties and out-patients, whilst 258 beds are constantly filled by in-patients. Its work is continually increasing. In order to meet the growing demands, a new Children's Department, with open-air playground, and a new Maternity Department have been equipped and occupied within the last twelve months. The cost of this forward movement has been paid. Not a shilling is owing, but—and it is a very big but—funds are urgently needed to meet the increased yearly expenditure. If every reader will send five shillings we should have no anxiety about the coming year. Gifts will be thankfully received by the House Governor, Charing Cross Hospital, Strand, W.C. 2.

**CITY OF LONDON HOSPITAL FOR CHEST DISEASES.**—Nearly 1 in 10 die annually from consumption and 1 in 7 from heart disease. The work of this hospital in combating these diseases needs steady support, especially in view of the fact that the war increased the suffering from consumption. Help is required in order to meet an increase in expenditure of £15,000 per annum. The Secretary is Mr. George Watts, City of London Hospital for Chest Diseases, Victoria Park, E. 2.

**GUY'S HOSPITAL.**—January 6th, 1925, will see Guy's Hospital 200 years old, and the Treasurer is endeavouring to celebrate this birthday by collecting £200,000 to pay off the debt of the Hospital and Medical School. Guy's Hospital has only made one previous big public appeal in the last two hundred years, although its endowed income is only about a third of its expenditure, and in consequence it is now making its great bicentenary appeal and asking all its friends to give and help to their utmost. The hospital has nearly 10,000 in-patients a year and nearly half-a-million out-patient attendances. The economy of its administration is best emphasized by the figures published by the King Edward's Fund. It has the cheapest cost per head per out-patient of all the twelve great London Hospitals with Medical Schools and the cheapest but two for maintenance per bed. All cheques should be made payable to the Treasurer, Guy's Hospital, S.E. 1.

**KING'S COLLEGE HOSPITAL.**—During the year 1924 forty more beds have been made available for general patients, including sixteen specially assigned to patients who have met with accidents. A special appeal has been issued to raise the additional income required to maintain them. At the same time efforts have been made to reduce the debt, and by a special grant from the King's Fund, together with the cancellation of a private loan, the amount has been reduced from £77,000 at the beginning of 1924 to £41,000, so that the further contributions will enable the debt to be almost, if not completely, extinguished. In connection with the Nursing School a new departure has been made by the establishment of a Preliminary Training School, and it has been arranged to accept a certain number of pupils, who do not intend to be nurses, for a course of Domestic Arts. The Dental Department which was opened in the previous year has already attracted a large number of patients, and the Dental School has enrolled a number of pupils. All the Departments of the Hospital bear testimony to its popularity among the people of South London, and it only needs increased financial support to make the whole of the magnificent building available for the relief of the sick. Contributions should be sent to the Treasurer, at the Hospital, Denmark Hill, S.E. 5.

**LONDON HOSPITAL.**—The splendid work done by the London deserves and needs widespread support. The cost of its maintenance is tremendous—£250,000—but the public is aware that every penny subscribed is well and wisely used. Mr. E. W. Morris, the House Governor, will be grateful for subscriptions and donations, which should be sent to him at the Hospital, Whitechapel Road, E. 1.

**LONDON FEVER HOSPITAL.**—Established in 1802, the income is derived from voluntary sources, supplemented by patients' payments, which in the case of ward patients cover only a small proportion of the cost. During the year 1923, 508 patients were treated, all suffering from infectious diseases. Further help is needed to assist this, one of London's oldest charities, to carry on the work, of which the annual cost is about £25,000, and at least £10,000 of this sum must be collected in the New Year. Subscriptions and donations should be sent to Mr. Herbert J. Say, Acting Secretary, London Fever Hospital, Islington, N. 1.

**LONDON LOCK HOSPITAL.**—Over £6,000 is required by Christmas in order to carry on this work of national importance. Owing to the special ante-natal treatment available, 464 babies have been born free of venereal disease and thus given an opportunity of growing up strong and useful citizens. Donations should be sent to Mr. Henry J. Eason, F.C.I.S., London Lock Hospital, Harrow Road, W. 9.

**LONDON HOMŒOPATHIC HOSPITAL.** — Strong efforts have been made during the year by the London Homœopathic Hospital, Great Ormond Street, W.C. 1, with the support of its president, the Duke of York, to increase its annual subscription list from the present inadequate amount of £2,000 to £3,000 in order to meet the demands of a daily growing number of patients, totalling in round numbers, during this year, to some 58,000 out-patient attendances and 1,700 in-patients. Towards the £1,000 required, the hospital has already raised £600, and a strong appeal is now made for a quick and generous response in the limited time that now remains to complete the list of new subscribers for presentation to H.R.H. the Duke of York at the close of the year. The Secretary is Mr. E. A. Attwood, London Homœopathic Hospital, Great Ormond Street, W.C. 1.

**PRINCE OF WALES'S GENERAL HOSPITAL.**—Tottenham is one of the poorest districts of Outer London, and this institution and its Convalescent Home require annually £30,000 to maintain 215 beds. Over 30,000 patients are treated each year, and are received without subscribers' letters, the only passport to secure the services of the hospital being necessity. There is a debt on maintenance account of £15,000, and an urgent appeal is made for donations towards the liquidation of this sum, which should be forwarded to Mr. F. W. Drewett, F.C.I.S., J.P., Director, Prince of Wales's General Hospital, Tottenham, N. 15.

**THE QUEEN'S HOSPITAL FOR CHILDREN, HACKNEY ROAD.**—The outstanding need of this hospital is enlargement. Every department is worked up to the hilt. The institution is deservedly popular throughout the crowded districts that surround it, and it receives a great deal of support in the form of small contributions. Its expenditure has increased from £15,000 pre-war to about £31,000, and that of its branch, The Little Folks' Home, Bexhill, from £1,500 to £3,000. As its endowments only bring in £1,500 a year, it is naturally always in need of funds for maintenance. The struggle for existence has for the past nine years stood in the way of development, and a comprehensive scheme of enlargement, which early in 1914 had received the blessing of King Edward's Hospital Fund, has had to be put aside. The present accommodation for in-patients is limited to 170 beds (134 at Hackney Road and 36 at the branch establishment at Bexhill). With 40,000 children passing through its out-patient and casualty departments in the year (and an attendance of over 100,000 a year) these beds are, of course, insufficient. Lord William Cecil, the Chairman, and his Committee are asking for £20,000 this year to pay arrears and generally to improve their hospital's financial position.

**ROYAL FREE HOSPITAL.**—To extend the existing buildings on an adjoining site recently presented to the hospital, £200,000 is required ; the intention of the authorities being to provide extended accommodation for the treatment of patients as well as for undergraduate and post-graduate medical education. Grants and the allocation of funds for this object are specially sought. All contributions should be sent to the Secretary, Mr. Reginald R. Garratt, Royal Free Hospital, Gray's Inn Road, W.C. 1.

**ROYAL NORTHERN GROUP OF HOSPITALS.**—This group comprises the Royal Northern Hospital (Holloway), the Royal Chest Hospital (City Road), and Hospital of Recovery at Southgate and a Convalescent Home at Clacton. In spite of rigid economy, and the closing of seventy beds, the accumulated debt is now £65,000, and the sick poor of North London are thereby deprived of much help and are suffering untold hardship. Assistance is needed at once, and needed badly, and the secretary, Mr. Gilbert G. Panter, Royal Northern Hospital, Holloway, N. 7, will be most grateful for contributions.

**ST. GEORGE'S HOSPITAL.**—Thirty-eight thousand patients are treated annually at this hospital, in addition to the 1,150 received at the recovery branch at Wimbledon, and the committee most urgently appeal for Christmas donations to help towards the reduction of the deficit on the current year's work. Contributions are also most earnestly desired for the new Extension of the Out-Patient Department, which has just been opened and which has increased the accommodation very materially. The cost of this has been £16,150, and any amount, large or small, will be thankfully received by Mr. James M. Churchfield, Secretary-Superintendent, St. George's Hospital, Hyde Park Corner, S.W. 1.

**ST. MARY'S HOSPITAL.**—The outstanding need of this well-known institution is the reduction of its formidable overdraft, which now stands at something like £30,000. Contributions to this end should be sent to the Secretary, Mr. W. Parkes, St. Mary's Hospital, Paddington, W. 2.

**SEAMEN'S HOSPITAL SOCIETY.**—Founded in 1821, and from that date until 1870 housed in a wooden man-of-war moored in the Thames off Greenwich, this Corporation treats merchant seamen of all nations. No letter of recommendation is required by a seaman and no patient is ever asked to pay for his illness—the Society being supported by voluntary contributions. The Society comprises six establishments : the "Dreadnought" Hospital, at Greenwich ; the Royal Albert Dock Hospital ; the Hospital for Tropical Diseases, Endsleigh Gardens, Euston ; King George's Sanatorium for Sailors, Bramshott, Hants. ; the Tilbury Seamen's Hospital, Tilbury ; the Angas Convalescent Home, Cudham, Kent. During 1923 the in-patients in all the establishments numbered 4,365, the new out-patients 16,837, making a total of 21,202. The inclusion this year of the Tilbury Hospital will add to these numbers and to the Society's annual expenditure. New wards, O.P. Department, Operating Theatre and Nurses' Home are being added to the Tilbury Hospital, which by June, 1925, will have accommodation for 50 in-patients and will therefore be adequate to serve the Tilbury district and the Docks in that station of the Port. The cost of this work—which is being carried out on the single storey plan advocated by Lord Dawson of Penn and other prominent physicians—will amount to £30,000, and only half of this sum is to hand. When laying the foundation stone of these buildings, the president, the Duke of York, said : "It is the duty and privilege of every citizen of the Empire to see that the seaman is nursed back to health when the misfortune of illness or accident falls upon him." It is confidently believed that a generous and patriotic public will assist this Society to carry on its truly national work. The Secretary is Sir James Michelli, C.M.G., Seamen's Hospital Society, Greenwich, S.E. 5.

**UNIVERSITY COLLEGE HOSPITAL.** — The annual income of this hospital falls short of the expenditure by some £14,000, and, in the absence of any emergency grant or other unexpected receipt, the hospital authorities will have seriously to consider the question of curtailing their work. Last year over 5,300 in-patients and over 76,000 out-patients were treated. Subscriptions and donations are earnestly requested to enable the Committee to carry on the work of the Hospital. They should be sent to Mr. J. Gerald T. Buckle, Secretary, University College Hospital, W.C. 1.

**VICTORIA HOSPITAL, CHELSEA.** — For the year 1922 the institution again proved to be one of the most economically managed children's hospitals in London. As a result of special grants from the combined appeal and the Voluntary Hospitals Commission, the hospital debt had been reduced to £4,238 by December 31. The ordinary income, however, is still insufficient to meet the yearly expenditure, and new annual subscriptions and donations are urgently required to place the institution on a better financial basis ; 180 beds have to be maintained, including 50 at the Victoria Home, Broadstairs, the hospital's seaside branch. Contributions should be sent to the Secretary, Mr. D. St. John Bamford, Victoria Hospital for Children, Tite Street, Chelsea, S.W. 3. A new feature of the institution is the private nursing home for children recently opened at 29, Tite Street, adjoining the hospital. A great opportunity is now offered to parents who wish their children to have the comforts of a private home combined with the treatment and other advantages of a leading London Hospital.

**WESTMINSTER HOSPITAL.**—The year 1924 will be memorable in the history of this institution as it marks the completion of the extensive alterations to the building and the reopening of the wards. The capacity of the hospital has been increased in response to the urgent need for more hospital accommodation in the metropolis and there are now 234 beds ; the out-patient department has been enlarged, a maternity ward and other special departments have been provided and important improvements have been made to the X-ray and electrical departments. The full cost of these alterations has been met, thanks to the most generous support of the public. Since the hospital was reopened the increased accommodation has been taxed to the uttermost. In view of the greater number of patients now receiving treatment the expenditure must show an increase, and to meet this the governors appeal for new donations and subscriptions, which should be sent to Sir Edward E. Pearson, Chairman, Westminster Hospital, Broad Sanctuary, S.W. 1.

---

### HOSPITALS WITH 50 AND LESS THAN 150 BEDS.

**THE CANCER HOSPITAL (FREE).**—This is the only hospital in London solely devoted both to treatment and to research into the causes of cancer. Cancer is now generally recognised as one of the most prevalent and dreaded of human diseases. The Cancer Hospital (Free) therefore has outstanding claims upon the consideration of the benevolent public ; and it is hoped that generous help may continue to be forthcoming to support this most worthy of institutions. Donations are also sought for the Reconstruction Building Fund, which is to provide new theatre accommodation and other improvements for the relief and benefit of the patients. Contributions should be sent to the Secretary, Mr. J. Courtney Buchanan, C.B.E., The Cancer Hospital (Free), Fulham Road, S.W. 3.

**CHELSEA HOSPITAL FOR WOMEN.**—The building of the greatly needed Nurses' Home is making good progress. A sum of £14,000 is still required. This work is supported by the King Edward's Fund, and, when completed will free for patients some forty-five beds in the hospital now being used for the staff. It will be possible to use some of these beds for a class of patients for whom there is all too little provision—viz., those who through reduction of income can no longer pay the charges of nursing homes. Contributions towards the Building Fund or towards daily maintenance will be gratefully received by the Honorary Treasurer or by the Secretary, Mr. Herbert H. Jennings, at 44, Arthur Street, Chelsea, S.W. 3.

**CITY OF LONDON MATERNITY HOSPITAL.**—The oldest maternity hospital in the Kingdom, trains annually nearly 100 midwives and maternity nurses. The national importance of this work becomes obvious when it is realised that nearly 75 per cent. of all confinement cases are attended by midwives. The Hospital's Antenatal Department corrects abnormal tendencies in numbers of cases, and its Child Welfare Department ensures to the babies a fair start in life. £15,000 is urgently needed to free the Hospital from debt and save the heavy charge for interest. Donations should be sent to the Secretary, City of London Maternity Hospital, City Road, E.C. 1.

**CLAPHAM MATERNITY HOSPITAL.**—This hospital though not in debt, earnestly appeals for Christmas donations or new subscribers to maintain and increase its usefulness. It is staffed by medical women, and its Hon. Secretary is Miss M. Ritchie, Clapham Maternity Hospital, S.W. 4.

**EVELINA HOSPITAL FOR CHILDREN.** — "The Evelina" is the largest hospital exclusively for children in the whole of south and south-east London, and serves several thickly populated and very destitute districts; it is for children of the poor only. Unfortunately, owing to its position, it is rarely seen by the "well-to-do" and suffers accordingly. A large sum is owing to the bankers, and contributions are needed to clear off the deficit; this year the expenditure has been unusually heavy, as the hospital has been entirely repainted and renovated, and several important improvements and alterations have at the same time been carried out. The total expenditure has been about £3,000. All of this work was long overdue and had been delayed owing to the serious scarcity of funds, coupled with the war conditions. The ordinary expenditure is over £12,000, but the assured income falls far short of this sum. and about £8,000 has to be raised annually in voluntary contributions. The Secretary is Mr. H. C. Standiland Smith, Evelina Hospital for Children, Southwark, S.E. 1.

**EVERSFIELD CHEST HOSPITAL, ST. LEONARDS.**—A mortgage debt of £1,200, which the Committee has decided must be liquidated, encumbers this Hospital, well known for its excellent work. Many of the male patients are "war-broken" men who contracted consumption while defending the country. The Hospital has therefore a special claim upon public sympathy, and, since the debt has been reduced from £4,500, it would be particularly grievous should friends fail speedily to clear off this remaining sum so that the institution may go forward unfettered. Donations should be sent to the Secretary.

**HAMPSTEAD GENERAL AND NORTH-WEST LONDON HOSPITAL.**—The expenditure of the Hospital has been reduced to £22,000, the minimum that can now be anticipated. Ordinary sources are producing an income of about £19,000 a year, which includes voluntary contributions by patients amounting to £4,500. The Council appeal to those who have not hitherto realised their obligations to the Hospital to contribute something each year towards the deficiency and thus enable them to carry on the work unhampered by debt, which now amounts to £6,000. The fact should not be lost sight of that the expenditure referred to makes no provision for those improvements and extensions which the advances of medical science and the increasing needs of the community demand. Contributions may be sent to the Secretary, Mr. Harold Wigg, Hampstead General and North-West London Hospital, Haverstock Hill, N.W. 3.

**LONDON TEMPERANCE HOSPITAL.**—The rebuilding and extension are now nearly completed (at a cost of £38,000), with the sum of £18,000 still needed. The hospital has been brought into line with modern requirements, and with its 135 beds and a large out-patients' department it is carrying on a great work of healing. All donations should be sent to the Chairman, Temperance Hospital, Hampstead Road, N.W. 1.

**METROPOLITAN HOSPITAL.**—A general hospital, with 137 beds, which receives its patients from one of London's poorest districts. Annual income from investments less than £1,000, leaving approximately £26,000 to be collected each year from voluntary sources. In addition it is imperative that the nursing staff be provided without delay with a suitable home to replace the deplorable quarters they now occupy. Contributions to either fund would be most welcome. Bankers: Glyn, Mills and Co., Lombard Street, E.C.; Secretary and House Governor: Mr. Herbert F. Rutherford, Metropolitan Hospital, Kingsland Road, E. 8.

**MILLER GENERAL HOSPITAL FOR SOUTH-EAST LONDON.**—The extension of this hospital, which has been under consideration for some time, has now been definitely decided upon, and it is hoped that it will be possible to start building operations in the spring of next year. The part of the scheme to be proceeded with is the first section of a much-needed nurses' Home and one ward block, which will contain 3 wards of 26 beds each—one of which will be "the Borough of Deptford's War Memorial Children's Ward." The site has been paid for and about £20,000 is in hand, but a large further sum will be required. The Secretary is Mr. H. A. Bone, Miller General Hospital, Greenwich Road, S.E. 10.

**MOUNT VERNON HOSPITAL FOR TUBERCULOSIS.**—At the out-patients' department, Fitzroy Square, W., this Hospital treats all deserving persons suffering from tuberculosis and diseases of the lungs and heart, and at the Hospital at Northwood patients are admitted on a contributory basis. The Secretary, Mr. W. J. Morton, earnestly appeals for subscriptions and donations to carry on the good work which this institution is doing on behalf of the suffering poor. All help of a financial character should be addressed to him at the Mount Vernon Hospital, 7 Fitzroy Square, W. 1.

**QUEEN CHARLOTTE'S MATERNITY HOSPITAL.**—Nearly 2,000 women are received into the wards of this institution every year, and over 2,000 are attended by the hospital midwives and nurses in their own homes. In addition, some 5,000 patients attend the ante-natal and child welfare department, their total attendances numbering 12,213. In the midwifery training school about 150 students are trained in midwifery annually, and nearly 200 woment are trained as midwives and maternity nurses. Great efforts have been made to free the Hospital of debt, but nearly £17,000 is still required. Moreover, the much needed enlargement of the Hospital, which was about to be commenced on the outbreak of war, has had to be postponed until more prosperous times. Donations will be gratefully received by the Secretary, Mr. Arthur Watts, at the Hospital, Marylebone Road, N.W. 1.

**QUEEN MARY'S HOSPITAL FOR THE EAST END.**—This Hospital appeals to all interested in the work of looking after the sick and suffering. It serves a great part of the East-End of London with a teeming population, and is situated in one of the largest boroughs in England. Contributions should be sent to Major Raphael Jackson, Secretary, Queen Mary's Hospital for the East End, Stratford, E. 15.

**ST. MARK'S HOSPITAL FOR CANCER.**—The Committee of Management earnestly appeal for contributions towards £15,000 required for the enlargement of the Hospital to cope with the increasing number of patients requiring alleviation from their sufferings. Additional wards, a new and more up-to-date out-patients' department, and larger cancer research laboratories are urgently required. Donations will be received by the Secretary, St. Mark's Hospital, City Road, E.C. 1.

**ST. PETER'S HOSPITAL.**—A sum of £2,500 is being asked for to improve the sleeping quarters of the domestic staff and to provide and equip an X-ray Department. Contributions should be sent to Mr. Irwin H. Beattie, Secretary, St. Peter's Hospital, Henrietta Street, Covent Garden, W.C. 2.

**SAMARITAN FREE HOSPITAL FOR WOMEN.**—The President, Princess Arthur of Connaught, appeals on behalf of this Hospital, which is greatly in need of funds. Through financial embarrassment, the Committee of Management have been unable to keep the Hospital in a safe condition. The structural defects, both external and internal, have become so dangerous that the architects advised the Committee that if nothing were done and we experienced a heavy winter, the safety of the Hospital would be in jeopardy. The total estimated cost of the necessary repairs and decorations amounted to £4,670. In view of the report of the architects, any further delay would have been fatal, and in order to meet this heavy expenditure the whole of the realisable investments of the Hospital have been sold. The effect of the loss of £5,000 of the investments will be sadly felt, for the dividends thereon form the only reliable source of income. Princess Arthur, having worked in the operating theatre and wards, testifies as to the splendid results obtained and earnestly solicits assistance towards the funds which are so urgently needed.

**SOUTH-EASTERN HOSPITAL FOR CHILDREN, SYDENHAM.**—In maintaining efficiently its 60 cots, in which over 500 children are nursed in the year, this Hospital has incurred a debt of over £2,000, and an appeal is made for donations to free the institution from this liability. In the out-patients' department, which has now been entirely rebuilt, the attendances number over 30,000 annually. Funds are urgently needed to carry on this work, and donations should be sent to Mr. W. Mason, Hon. Secretary, 88 Dacres Road, Forest Hill, S.E. 23.

**SOUTH LONDON HOSPITAL FOR WOMEN.**—A new wing affording accommodation for thirty-six additional surgical cases was opened by the Duchess of York in May last. The beds available for patients now number eighty-nine in general wards and twenty-four in private wards. It is anticipated that this extension will entail an additional expenditure of £6,000 per annum. Especial appeal is made for new annual subscriptions and donations to meet this increased cost, also for donations to the Samaritan Fund, which is in great need of assistance. These should be sent to the Secretary, South London Hospital for Women, South Side, Clapham Common, S.W. 4.

### HOSPITALS WITH LESS THAN 50 BEDS.

**BELGRAVE HOSPITAL FOR CHILDREN.** — The William Shepherd Wing was opened by the Prince of Wales in February last providing two additional wards of twelve cots each. It is estimated that the opening of this wing will increase the total cost of maintenance by £2,500 per annum. The committee appeal most earnestly for increased support to enable them to keep these greatly needed cots at the service of South London's sick children. The nurses are now uncomfortably housed in three separate places, and the committee appeal for £25,000 to enable them to build the west wing in order that the whole of the nursing staff may be more suitably accommodated under one roof.

**MILDMAY MEMORIAL HOSPITAL.**—This hospital is in urgent need of donations to enable it to reconstruct its small and inadequate operating theatre and also to erect a very necessary electric lift. One thousand one hundred pounds is required for this purpose, of which £300 only is at present available. Donations should be sent to the Secretary, Miss E. D. Papprill, Mildmay Memorial Hospital, Newington Green Road, N. 1.

**PADDINGTON GREEN CHILDREN'S HOSPITAL.**—The hospital has 46 beds, and a Convalescent Home at Slough with 24 beds. The income for 1923 from all sources was £10,865, and the expenditure £12,205. Some 870 in-patients received treatment during the year, in addition to 12,122 new out-patients, while the total out-patients' attendances numbered about 43,700. The committee of management urgently appeal for new subscriptions, donations and legacies, to enable them to discharge the very heavy debt of £15,000 owing to bankers. Contributions will be thankfully received by the Secretary, Mr. James A. Hamlin, at the Paddington Green Children's Hospital, W. 2.

**ROYAL EYE HOSPITAL.**—This is the only eye hospital in South London, and serves the needs of a very large and populous district. It has 22,000 new out-patients, more than 50,000 attendances annually, and its expenditure exceeds income by £1,000. Funds are urgently needed and should be sent to the Secretary, St. George's Circus, Southwark, S.E. 1.

**ROYAL WESTMINSTER OPHTHALMIC HOSPITAL.**—Founded 108 years ago, this Hospital is rapidly increasing its work of treating sufferers from diseases or injuries to the eye, who attend from all parts of the Metropolis and the provinces, the yearly attendances being upwards of 35,000. Funds are greatly needed, especially in view of the fact that 84 per cent. of the required annual income must be obtained from voluntary sources. Benefactions will be gratefully acknowledged by the Secretary, Capt. J. H. Johnson, King William Street, West Strand, W.C. 2.

**ST. JOHN'S HOSPITAL FOR DISEASES OF THE SKIN.**—Founded in 1863, this Hospital is the largest of its kind in London, dealing with 1,000 cases a week. Like other institutions, it is suffering from the present high prices of all commodities, and the board of management of this thoroughly deserving and necessary charity appeal earnestly for help. The recently formed London School of Dermatology is the recognised centre of dermatological teaching in London, its staff of Lecturers consisting of the Dermatologists from the twelve principal Medical Schools. Cheques will be gratefully received by the Treasurer, Captain E. C. Eric Smith, M.C., 49 Leicester Square, W.C. 2, or by the Secretary, at the same address.

### GENERAL CHARITIES.

**CHRISTMAS COMFORTS FOR PENSIONER PATIENTS.**—The Order of St. John and the British Red Cross Society have arranged, through the Ministry of Pensions, as in previous years, to provide additional comforts, such as cigarettes, tobacco, chocolate and games, for the men actually in Ministry Hospitals on Christmas Day. There are, however, ex-Service pensioners who will be patients in other hospitals as a result of their war disabilities, and the Joint Council of the two bodies is endeavouring to get in touch with these hospitals, so that no eligible cases in hospital on Christmas Day may fail to receive the same gifts as those in Pensions Hospitals.

**DR. BARNARDO'S HOMES.**—The Charter of the Homes is "No Destitute Child Ever Refused Admission." The one and only qualification, therefore, for admission is destitution. In 1923, 13,276 boys and girls were dealt with; 1,279 being permanently admitted, and there are to-day 7,308 children under the care of the Homes. Some 28,681 boys and girls have been emigrated within the Empire, of which 98 per cent. are doing well. The institution comprises 156 separate branches and households, and the work formerly carried on at Her Majesty's Hospital, Stepney Causeway, is now transferred to the Australasian Hospital at the Girls' Village Home, Barkingside, Essex, where there are 1,400 girls always in residence; and to the John Capel Hanbury Hospital at the Boys' Garden City, Woodford Bridge, Essex, where are housed over 700 boys. An earnest appeal is made for help, and will be welcomed at the Head Offices and Chief Ever-Open Door at 18-26 Stepney Causeway, E. 1.

**IMPERIAL CANCER RESEARCH FUND.**—This society is carrying on a very important work in the interest of suffering humanity, and it is hoped that its investigations into the causes of cancer, and its efforts to find a cure for this dreaded disease, may not be hampered for want of adequate funds. Donations and subscriptions should be sent to the Honorary Treasurer, Imperial Cancer Research Fund, 8-11 Queen Square, Bloomsbury, W.C. 1.

**MANCHESTER AND SALFORD MEDICAL CHARITIES.**—This Fund is appealing on behalf of the twenty-four hospitals and medical charities of Manchester and Salford. These form a suitable centre of healing, and the ordinary year's expenditure is as great as £335,000. The deficit of the year is £44,000, and all contributions should be sent to the Secretary, Capt. T. Spencer, 49 Deansgate, Manchester.

**MENTAL AFTER-CARE ASSOCIATION.** — Seven thousand men and women are discharged every year from our mental hospitals, and a number of these have no home, no friends and no financial resources. It is to help these unfortunate people that this Association exists, since, unaided, many relapse and become permanent burdens on the rates or sink into abject poverty. The Association, whose secretary is Miss Vickers, Church House, Westminster, S.W., acts as a Labour Bureau, finding not only occupation, but often the means necessary to undertake it, by clothing, tools and in other ways.

